# Intestinal epithelial cell apoptosis and loss of barrier function in the setting of altered microbiota with enteral nutrient deprivation

**DOI:** 10.3389/fcimb.2013.00105

**Published:** 2013-12-23

**Authors:** Farokh R. Demehri, Meredith Barrett, Matthew W. Ralls, Eiichi A. Miyasaka, Yongjia Feng, Daniel H. Teitelbaum

**Affiliations:** Section of Pediatric Surgery, Department of Surgery, University of Michigan Health SystemAnn Arbor, MI, USA

**Keywords:** small intestine, parenteral nutrition, epithelial barrier function, epithelial cell apoptosis, epithelial call proliferation, microbiome

## Abstract

Total parenteral nutrition (TPN), a commonly used treatment for patients who cannot receive enteral nutrition, is associated with significant septic complications due in part to a loss of epithelial barrier function (EBF). While the underlying mechanisms of TPN-related epithelial changes are poorly understood, a mouse model of TPN-dependence has helped identify several contributing factors. Enteral deprivation leads to a shift in intestinal microbiota to predominantly Gram-negative Proteobacteria. This is associated with an increase in expression of proinflammatory cytokines within the mucosa, including interferon-γ and tumor necrosis factor-α. A concomitant loss of epithelial growth factors leads to a decrease in epithelial cell proliferation and increased apoptosis. The resulting loss of epithelial tight junction proteins contributes to EBF dysfunction. These mechanisms identify potential strategies of protecting against TPN-related complications, such as modification of luminal bacteria, blockade of proinflammatory cytokines, or growth factor replacement.

## Parenteral nutrition: a necessary risk

Maintaining appropriate nutrition in patients is a clinical necessity that is at times difficult to accomplish. Particularly devastating complications are seen in the perioperative period where malnourishment increases complications, delays wound healing, decreases immune function, prolongs hospitalizations, and lowers patient quality of life (Peter et al., 2005; Martindale et al., 2013). The ideal and most physiologic form of nutrition is via the gastrointestinal tract (Moore et al., 1992; Braunschweig et al., 2001; Mirtallo et al., 2004; Peter et al., 2005; Zaloga, 2006). Unfortunately, due to mechanical, functional, or postoperative deficits, some patients are unable to use their intestinal tract for nutritious gain. For these patients who are enterally deprived, total parenteral nutrition (TPN) is an alternative method of nutrition which provides nutrients and calories intravenously (Abunnaja et al., 2013). According to the United States Healthcare Cost and Utilization Project, greater than 352,000 patients received TPN in the United States in 2010 (Pfuntner et al., 2006). It thus serves as a life sustaining treatment for many patients.

A recent adjunct to the nutritional armamentarium, TPN has been used regularly for patients since the mid-twentieth century. Through groundbreaking work by Rhoads and colleagues in the 1960s, challenges regarding access and formulation were overcome and TPN was able to be prescribed to those who would have previously had no means of nutritional access (Dudrick et al., 1968). Efforts to optimize the composition, administration, and patient selection criteria for TPN have since continued, as codified in recent guidelines issued by the American Society for Parenteral and Enteral Nutrition (Mirtallo et al., 2004).

TPN itself is a solution composed of amino acids, sugars, fat emulsions, electrolytes, vitamins, and trace elements (Zaloga, 2006). Due to its hypertonicity, it is recommended that central venous catheters are used for TPN delivery (Mirtallo et al., 2004). Assessment of the need for continued TPN utilization should be performed regularly as TPN, though life preserving, is not without significant risk. In fact, complications of TPN have been extensively described in the literature (The Veterans Affairs Total Parenteral Nutrition Cooperative Study Group, 1991; Moore et al., 1992; Buzby, 1993; Braunschweig et al., 2001; Peter et al., 2005; Heneghan et al., 2013). Blood stream infection, hepatic dysfunction, metabolic derangements, bacterial translocation, immunologic compromise, and enterocyte atrophy are well known sequelae of TPN administration. In a historic multi-hospital Veterans Affairs study, Buzby et al. (Dudrick et al., 1968) found patients with mild malnutrition received no benefit from TPN and suffered from infectious complications “not explained by the presence of a catheter”—including pneumonia, urinary tract infection, and surgical site infection. In a recent study, Casaer et al. expanded upon these findings, demonstrating not only a lack of benefit with early administration of TPN in critically-ill patients, but in fact poorer outcomes compared to later and more judicious initiation of TPN (Casaer et al., 2011). Such complications have led to cautious administration of TPN and utilization of enteral feeding whenever possible (Braunschweig et al., 2001).

## Physiologic and immunologic changes with TPN in a mouse model

**Figure 1:**
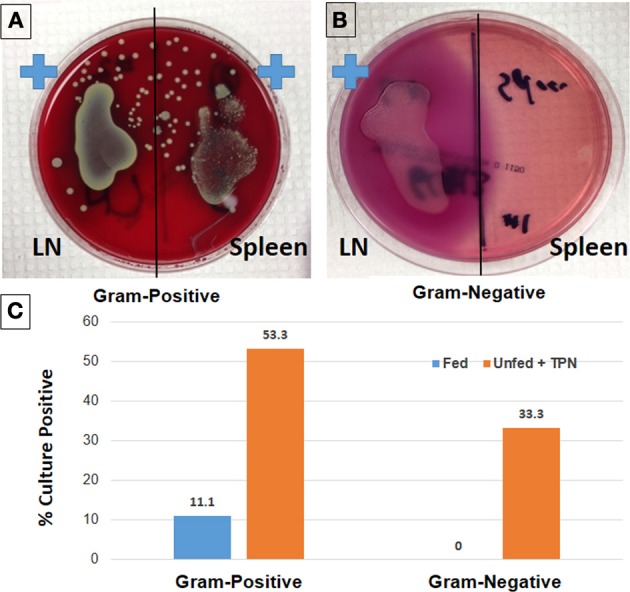
**Loss of epithelial barrier function with TPN leads to bacterial translocation and sepsis**. Lymph node (LN) and spleen isolates from TPN-dependent mice were incubated on Columbia-CNA agar **(A)** to isolate Gram-positive bacteria. Similarly, culture on MacConkey agar demonstrates Gram-negative bacteria from TPN-dependent LN isolates **(B)**. Rates of bacteremia are significantly greater in TPN-dependent vs. fed mice **(C)** (Sun et al., 2006).

**Figure 2:**
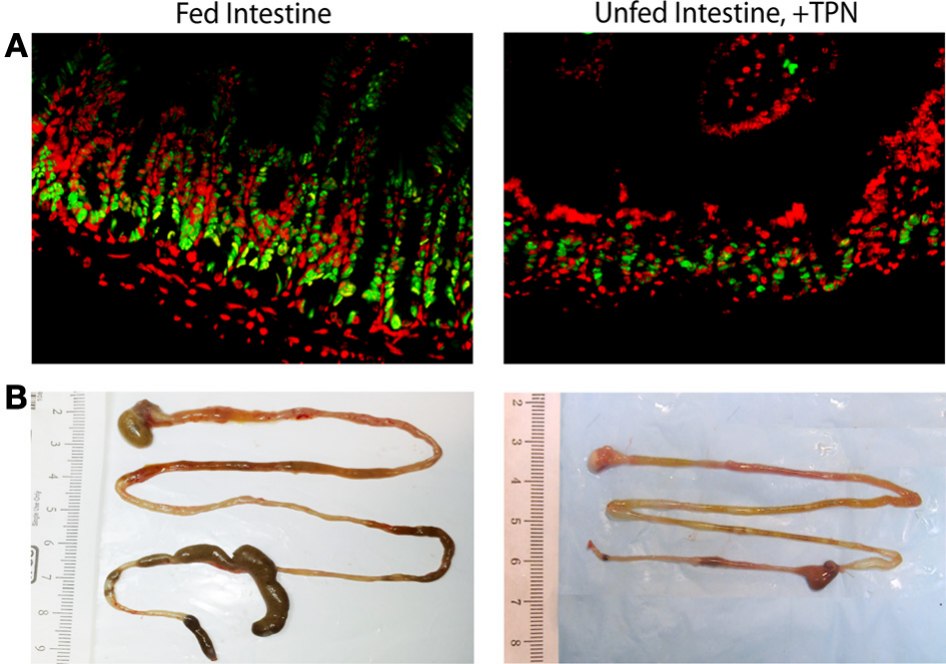
**Epithelial and whole-bowel changes with TPN**. Unfed bowel demonstrates decreased epithelial cell proliferation (green = PCNA, proliferating cells; red = DAPI, all nucleated cells) compared to fed intestine **(A)**. Representative images of harvested mouse intestine demonstrate decreased length with TPN-dependence **(B)**.

While the etiology of the increased rate of infectious complications with TPN is not fully established, it is known that many of the organisms responsible for these infections predominantly originate from enteric microbiota, suggesting a TPN-associated loss of intestinal epithelial barrier function (EBF) (Feng et al., 2012). TPN-induced changes allow translocation of endotoxins and enteric microbiota across the epithelial barrier, leading to endotoxemia, bacteremia and sepsis (Alverdy et al., 1988) (Figure 1). A mouse model of TPN has allowed the identification of regulatory pathways which are involved in these TPN-induced physiologic changes. In this model, mice receive TPN via an internal jugular catheter and no enteral nutrition. These are compared to control mice which also have an intravenous catheter place, but receive only saline (electrolyte solution) and are allowed enteral food. Using such a model, our laboratory and others have identified several contributing factors which drive this diminished EBF with TPN, such as loss of local growth factors, increased levels of pro-inflammatory mucosal cytokines, and alterations in intraluminal microbiota. These TPN-induced changes result in a pro-inflammatory state within the intestinal mucosa, leading to villous atrophy, an increase in epithelial cell (EC) apoptosis, and a decrease in EC proliferation (Wildhaber et al., 2002)—as also demonstrated by the reduction in overall length of the small and large bowel (Figure 2).

### Inflammatory cytokine dysregulation: IFN-γ and TNF-α

Inflammatory cytokines linked to loss of EBF include interferon gamma (IFN-γ) and tumor necrosis factor alpha (TNF-α), both of which are increased in animal models of TPN-dependence. IFN-γ is a cytokine which, when administered *in vitro* to a T84 epithelial monolayer, results in the loss of tight junction integrity (Madara and Stafford, 1989). This effect is prevented by pretreatment with transforming growth factor-β1 (Planchon et al., 1994). TPN-dependent mice have been shown to have a greater than three-fold increase in IFN-γ mRNA expression, with subsequent EBF breakdown. This loss of EBF is mitigated via blockade of IFN-γ signaling using IFN-γ-knockout mice (Yang et al., 2002, 2003a).

Similarly, our work has also shown that TNF-α expression is increased in response to enteral nutrient deprivation and TPN administration (Feng and Teitelbaum, 2012). This increase is associated with mucosal atrophy and loss of EBF. Using a combination of TNF-receptor-1 (TNF-R1) and TNF-R2 knockout mice, blockade of TNF signaling prevents mucosal atrophy with TPN, but blockade of both receptors are needed to prevent the loss of EBF associated with TPN (Feng and Teitelbaum, 2012). TNF-receptor-dependent downstream mediators include nuclear factor-κB (NF-κB) and myosin light chain kinase (MLCK). TNF-receptor activation leads to up-regulation of MLCK with the activation of myosin light chain (MLC) to phospho-MLC (Feng and Teitelbaum, 2013). Intestinal epithelial cell (IEC) MLCK is known to mediate TNF-α-induced modulation of the intestinal epithelial tight junction barrier (Ye et al., 2006). The EBF is maintained by the integrity of a series of paracellular tight junctional proteins, including zonula occludens 1 (ZO-1), ZO-2, junctional adhesions, occludin as well as a large family of claudins. With the phosphorylation of MLC, an activation of actin and myosin contraction occurs on the IEC apical surface, with the dissociation of ZO-1 from the tight junction, an internalization of occludin, and a resultant deterioration of barrier function (Chen et al., 2012). This process can be progressive, as TNF-α will drive the downstream activation of NF-κB, which is responsible for initiating an inflammatory amplification cascade (Barnes and Karin, 1997). Another important regulatory role of TNF-α is the mediation of IEC apoptosis. Signaling of TNF-α via the TNF-R1 and TNR-R1 receptors are principal pathways for IEC apoptosis (Feng and Teitelbaum, 2012). Interestingly, both MLCK and NF-κB are up-regulated with TPN, and these changes are prevented with TNF-α blockade (Feng and Teitelbaum, 2013).

### Diminished growth factor and immune regulator signaling

In addition to mediating inflammation and apoptosis, TNF-α has recently been shown to play a critical role in enterocyte survival and regulation of IEC proliferation (Edelblum et al., 2008). A potent mediator of EC proliferation, epidermal growth factor (EGF) signaling is dependent on both ErbB-1 receptor and TNF-α signaling pathways (McElroy et al., 2008; Yamaoka et al., 2008). With TPN administration, EGF signaling is diminished secondary to both TNF-α dysregulation along with decreased ErbB-1 expression. Correction of this TPN-induced imbalance via exogenous EGF or through a blockade of TNF-R1 was shown to partially reverse the pro-apoptotic effects of excessive TNF-α signaling (Feng and Teitelbaum, 2012). Two other mucosal growth factors, keratinocyte growth factor and glucagon-like peptide 2, have also been shown to be diminished with TPN. These two growth factors, like EGF, play a role in maintenance of EBF and their diminished expression contributes to EBF loss (Feng et al., 2012).

The regulation of IEC integrity is also linked to the intraepithelial lymphocyte (IEL)-derived anti-inflammatory interleukin 10 (IL-10), which is a master regulator of the mucosal immune system (Duell et al., 2012). Knockout mice who fail to produce IL-10 demonstrate increased epithelial permeability (Madsen et al., 1999), resulting in a colitis model (Berg et al., 2002). In TPN-dependent mice, a significant decline in IEL-derived IL-10 is seen along with an associated decrease in EBF (Sun et al., 2008). By administering exogenous IL-10, the TPN-associated decline in intestinal barrier function is attenuated; suggesting that loss of IL-10 contributes to TPN-induced epithelial barrier dysfunction.

### EBF breakdown is modulated by decreased p-Akt signaling

The decrease in growth factor signaling with TPN leads to a downstream decrease in phosphatidylinositol 3-kinase (PI3K)/p-Akt signaling. PI3K/p-Akt signaling is known to play a key role in cell cycle progression and preventing apoptosis (Chang et al., 2003). With TPN, p-Akt activity diminishes in ECs, with an associated loss of EC proliferation and increased apoptosis (Feng et al., 2009). Using an Akt-activating peptide T-cell lymphoma-1 (TCL1) conjugated to a transactivator of transcription peptide sequence (TAT), a significant increase in p-Akt abundance was achieved in TPN-dependent mice, along with prevention of the loss of EC proliferation and increased apoptosis seen with TPN (Feng et al., 2010). This demonstrates the central importance of p-Akt signaling in maintaining EBF integrity.

Thus, TPN causes increased levels of inflammatory cytokines TNF-α and IFN-γ, along with decreased production of inflammatory regulator IL-10 and growth factor EGF, which together lead to altered IEC survival and proliferation. In addition, TPN-dependence appears to significantly diminish EBF. The components that make up EBF include the synthesis and release of mucus from goblet cells, transcytosis of dimeric secretory IgA [which is also lost with TPN (Fukatsu and Kudsk, 2011)], intraluminal movement of water, and the physical integrity of the epithelium itself (Clayburgh et al., 2004). Breakdown of this barrier can lead to the translocation of intestinal microbiota and/or endotoxin, which are thought to contribute to TPN-related sepsis (Kristof et al., 2011).

TPN-dependence appears to decrease the integrity of the epithelial junctional protein apparatus, which is a critical component of EBF. Junctional proteins include ZO-1, which cross-links the E-Cadherin/catenin complex and the actin cytoskeleton (Itoh et al., 1997), as well as occludin and the family of claudins, the latter of which has been found to be altered in Crohn's disease (Zeissig et al., 2007). In TPN-dependent mice, all of these junctional proteins have been found to have a significant reduction in abundance compared to enterally-fed mice (Sun et al., 2008). Expression of these proteins is linked to EBF, which can be reflected via measurement of the transepithelial potential difference and resistance using an Ussing chamber (Yang et al., 2003b). The previously described cytokine changes with TPN are also linked to junctional protein expression. For instance, exogenous IL-10 administration ameliorated TPN-induced loss of ZO-1, ZO-2, claudin-2, and occludin (Sun et al., 2008). Production of these proteins is also related to p-Akt signaling, as our work has shown that down-regulation of E-Cadherin expression in TPN-dependent mice is tightly related to a decrease in p-Akt activity (Feng et al., 2009). In addition, we demonstrated prevention of the TPN-induced loss of ZO-1 and E-cadherin expression when p-Akt activity is upregulated with TCL-1 administration. The loss in EBF is clearly multifactorial, however, as TCL-1 failed to prevent the loss of occludin expression or the decrease in transepithelial resistance with TPN (Feng et al., 2012). Interestingly, supplementation of TPN-dependent mice with intravenous glutamine, a critical amino acid, has been shown to preserve EBF and restore EC proliferation via prevention of loss of p-Akt abundance (Nose et al., 2010).

A key distinction in the mechanism of these TPN-induced changes is whether they are due to administration of the TPN solution itself or the lack of enteral feeding. To address this, we compared mice receiving TPN without feeding vs. TPN with partial (25%) oral feeding. This small amount of enteral nutrition prevented intestinal epithelial atrophy and the associated increase in proinflammatory cytokines while restoring normal tight junction function and EBF (Wildhaber et al., 2005). Thus, the lack of intraluminal nutrients appears to drive the pathophysiology of TPN-dependence.

## Changes in the gut microbiome with TPN

While the alterations in inflammatory cytokines and growth factors thus far described have a clear role in the physiologic changes seen with TPN, the mechanisms behind these alterations are unknown. An increasing body of evidence suggests that a driving force behind these changes is a drastically altered intestinal microbiome with associated immunologic changes. Normally composed of a diverse population of bacteria numbering up to 10^14^ colony-forming units, the intestinal microbiota play an essential role in host physiology via immune stimulation and regulation, digestion of carbohydrates otherwise unavailable to enterocytes, and production of key nutrients such as short-chain fatty acids (Salzman, 2010; Sekirov et al., 2010).

The interaction between host and microbiota is a complex relationship that is not completely understood. One of the critical functions played by intestinal microbiota is modulation of the host's immune system through interaction with lamina propria (LP) cells. A principal function of LP cells is the detection and monitoring of changes in the intraluminal environment (Novak and Bieber, 2008). Microbiota interact with LP cells via the toll-like receptor (TLR) pathway (Chichlowski and Hale, 2008; Ng et al., 2010). Several bacterial components act as ligands for TLRs, such as lipopolysaccharide (LPS), which is derived from Gram-negative bacteria and binds TLR4. TLR binding leads to downstream activation of NF-κ B signaling via a myeloid differentiation primary response gene 88 (MyD88) dependent pathway (Karrasch and Jobin, 2008; Abreu, 2010). Activation of NF-κ B, as described above, then mediates the expression of a number of proinflammatory cytokines.

**Figure 3:**
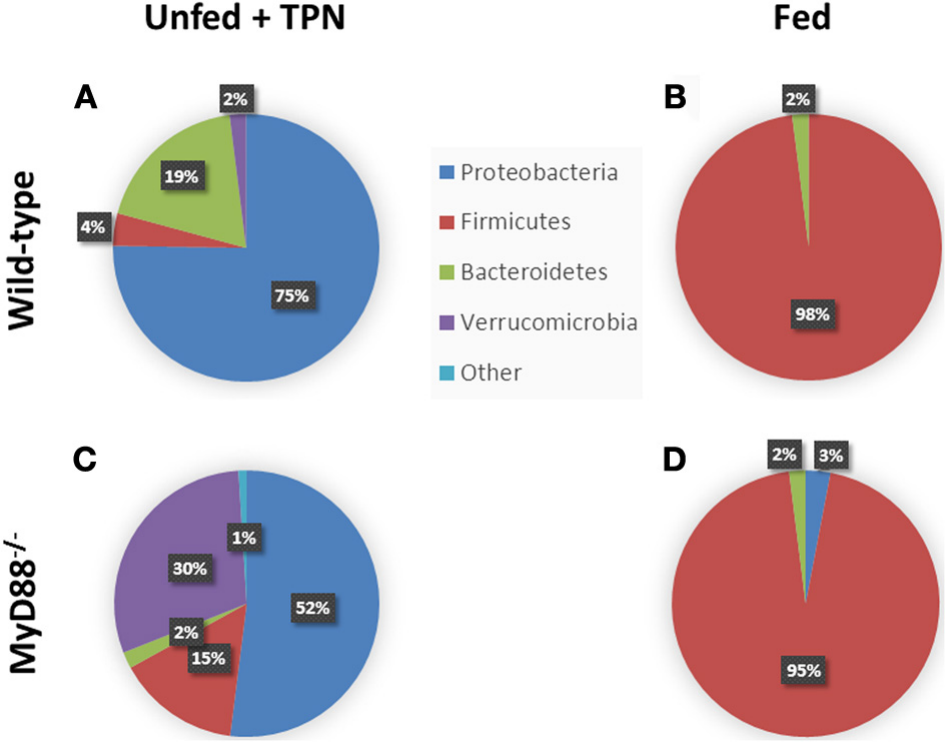
**Phylum-level changes in intestinal microbiota with TPN**. Enteric bacteria from TPN-dependent mice **(A)** demonstrate a relative increase in Proteobacteria and Bacteroidetes vs. fed mice **(B)**, where Firmicutes dominates. MyD88^−/−^ mice, with defective TLR signaling, demonstrate an additional expansion of Verrucomicrobia with TPN-dependence **(C)** vs. fed controls **(D)**. Akkermansia species predominate among the Verrucomicrobia. Adapted from Miyasaka et al. (2013).

While TPN supplies adequate nutritional needs systemically to the recipient, enteral deprivation puts the intestinal microbiota in an abrupt state of nutrient withdrawal. This has been shown to dramatically alter the makeup of small intestinal microbiota from a normally benign composition of predominantly Gram-positive Firmicutes to a Gram-negative Proteobacteria-dominated population. Additional phylum-level changes include increases in Bacteroidetes and Verrucomicrobia (predominantly Akkermansia) (Figure 3). This shift is associated with increased TLR signaling—specifically, up-regulation of TLR-2, 4, 7, and 9. The subsequent proinflammatory state within the LP is characterized by increased TNFα, IFN-γ, downstream NF-κB activation, and a marked loss in the LP T-regulatory (T_reg_) cell population (Miyasaka et al., 2013).

The deprivation of enteral nutrients available to intraluminal bacteria alters the selection pressure determining the dominant species of microbiota. In an environment of relative starvation, Proteobacteria have been shown to demonstrate resilience (Sinclair and Alexander, 1984), while Firmicutes establish themselves in the enterally-fed state (Costello et al., 2010). While these changes are characterized by a relative increase in Gram-negatives, there remains an undefined role for Gram-positive bacteria in TPN-dependence, given these organisms activate multiple TLRs (Patten and Collett, 2013) as well as apoptosis signaling (Ulett and Adderson, 2006). Besides the lack of luminal nutrients, host factors play a role in changing the microbial environment. For instance, TPN administration has been shown to lead to an increase in goblet cell numbers (Conour et al., 2002) and a decrease in Paneth cell function. Paneth cells, located adjacent to intestinal stem cells in crypts, interact with intestinal bacteria by secreting bactericidal proteins. TPN leads to decreased expression of Paneth cell-related antimicrobial proteins REGIII-g, lysozyme, and cryptdin-4. This leads to increased susceptibility to enteroinvasion by *E. coli* (Heneghan et al., 2013). These findings demonstrate a significant change in the host-microbiome relationship with TPN. An altered bacterial population develops in an environment with diminished mucosal defenses, contributing to continued EBF breakdown and septic complications.

## Blockade of MyD88 prevents the host response to an altered microbiome

To investigate the mechanism by which the altered microbiome contributes to TPN-related IEC changes, we have used TPN-dependent and chow-fed MyD88 knockout mice. Microbiota interact with host LP myeloid cells via TLR-signaling, much of which is mediated via the sub-cytoplasmic membrane protein, MyD88 (Abreu, 2010). Similar changes in gut microbiota have been observed in MyD88 knockout mice given TPN, with a shift from a Firmicutes-predominant community in fed mice to a Proteobacteria-dominant bacterial community in TPN-dependent mice. In this model, it indicates that the change in bacterial composition occurs independently of MyD88, supporting the theory that the lack of enteral nutrition itself drives the microbial changes. This is in contrast to a recent study in which the targeted deletion of MyD88 within the epithelium led to distinct changes within the mucosally-associated microbial population (Frantz et al., 2012).

The TLR signaling changes normally induced by this bacterial shift, however, are abrogated in the MyD88 knockout strain. Blockade of TLR signaling in these mice prevents sensing of the altered microbiota by the host LP cells. This leaves the mucosal immune response unchanged from that of fed mice. Pro-inflammatory cytokines TNF-α and IFN-γ are not upregulated, and activation of NF-κB is prevented. In addition, MyD88 blockade led to preservation of the small intestinal T_reg_ cell population, which is almost completely lost in wild-type TPN-dependent mice. Together, prevention of these inflammatory mucosal responses allows maintenance of EC proliferation, decreased apoptosis, and preservation of EBF (Miyasaka et al., 2013).

Interestingly, MyD88 knockout mice on TPN demonstrate an increase in Akkermansia species compared to wild-type TPN-dependent mice and fed mice (Figure 3). This may reflect the role of mucosal responses in modulating the intraluminal environment. By preventing the dominant Proteobacteria from signaling via TLRs, the inflammatory changes in the epithelium are avoided, and this may create an altered environment that is more favorable for this strain of bacteria (Miyasaka et al., 2013). A mechanism for this change may be due to an increase in acidomucins within the small bowel mucosa during the administration of TPN, the primary substrate for Akkermansia mucinophilia (Derrien et al., 2011). The complex interaction between the various microbial species, their luminal environment, and the intestinal epithelium continues to be explored.

## Evidence of barrier function loss and microbiota changes in humans

While the outcomes of decreased EBF and subsequent septic complications associated with TPN are well-documented in humans, the mechanisms described thus far have not been thoroughly explored in human intestine. In a study of 8 healthy volunteers who received TPN as an exclusive means of nutrition for 14 days, many of the intestinal morphologic and functional changes seen in animal models were reproduced, though to a lesser extent (Buchman et al., 1995). These changes included a decrease in mucosal thickness, increased villus cell count, and an increase in the urinary lactulose-mannitol ratio (indicating an increase in intestinal permeability). Mitotic index was not significantly diminished, however. While other studies in humans have failed to replicate increased sensitivity to intravenous LPS (Santos et al., 1994) or decreased EBF (Reynolds et al., 1997), these studies are based on a limited number of patients and lack a robust evaluation of changes in mucosal physiology.

A recent study has begun to elucidate whether a similarly significant change in gut microbial diversity occurs with enteral deprivation in humans (Ralls et al., 2013). Small bowel samples from 12 patients undergoing intestinal resection were collected and mucosa-associated bacteria were analyzed. As noted in other studies of human intestinal microbiota (Costello et al., 2012), a wide variability in microbial diversity was found within all groups. While an accurate characterization of the typical makeup of intestinal microbiota with TPN in humans was not possible, an interesting finding was that the level of microbial diversity appeared to be closely related to clinical outcome. Patients with low enteric bacterial diversity were significantly more likely to develop postoperative infection or intestinal anastomotic disruption (Ralls et al., 2013).

## Conclusion and future directions

**Figure 4:**
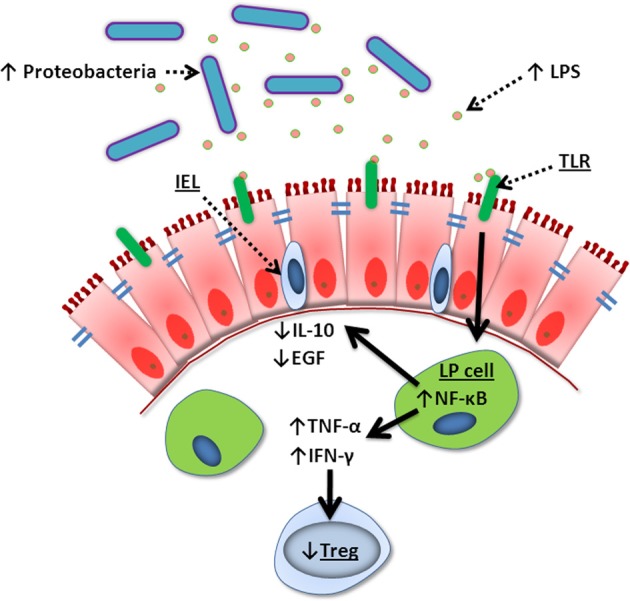
**Summary of TPN-induced epithelial signaling changes**. Lack of enteral nutrition leads to a change in luminal microbiota where Gram-negative Proteobacteria dominate. Lipopolysaccharide (LPS) derived from these bacteria signal lamina propria (LP) cells via Toll-like receptors (TLR), leading to increased NF-κ B transcription. This creates a pro-inflammatory state with increased TNF-α and IFN-γ, loss of T_reg_ cells, and decreased intraepithelial lymphocyte (IEL)-derived IL-10 and EGF. These changes lead to break down of tight junctions, loss of epithelial barrier function, bacterial translocation, and sepsis.

While TPN serves a life-sustaining purpose in patients who must remain deprived of enteral nutrition for a prolonged period, it is not without risk. Enteral deprivation leads to a drastically altered intestinal luminal environment, which allows for the dominance of aggressive microbiota such as Gram-negative Proteobacteria. Signaling factors such as LPS derived from these bacteria act through intestinal TLRs to stimulate an increase in pro-inflammatory mediators and a decrease in growth factors. These changes lead to a decrease in EC proliferation and increase in EC apoptosis. The final outcome is diminished EBF, characterized by a loss of epithelial tight junction proteins and increased mucosal permeability, with the subsequent septic morbidity associated with TPN (Figure 4).

A deeper understanding of the interaction between host and intestinal microbiota in the setting of enteric deprivation may provide novel strategies for preventing TPN-related complications. While evidence suggests that microbiota signal mucosal inflammation via TLRs using a MyD88 pathway, studies with more specific inhibition of certain TLRs may reveal that a subset of TLRs (i.e., TLR4, which binds LPS) may be primarily responsible for this process. In addition to understanding how epithelial physiology changes in this setting, it will be critical elucidate how luminal environment changes are tied to bacterial selection. Modifying this environment to select for more benign bacterial species may alleviate some of the deleterious changes seen with TPN. Finally, the altered cytokine profile produced by these changes may be alleviated pharmacologically. For example, growth factor replacement with exogenous EGF or antibody blockade of epithelial TNF-α may diminish TPN-related inflammation and loss of EBF.

Using a mouse model, significant gains have been made in the understanding of intestinal physiologic changes with fasting and TPN. Further human studies are needed to translate these findings to the patient. With a clear understanding of these changes, novel strategies to mitigate them may make TPN a safer option for the many patients who require it.

### Conflict of interest statement

The authors declare that the research was conducted in the absence of any commercial or financial relationships that could be construed as a potential conflict of interest.
